# A participatory approach to address within‐country cross‐border malaria: the case of Menoreh Hills in Java, Indonesia

**DOI:** 10.1186/s12936-021-03673-7

**Published:** 2021-03-06

**Authors:** Riris Andono Ahmad, Astri Ferdiana, Henry Surendra, Tyrone Reden Sy, Deni Herbianto, Theodola Baning Rahayujati, Dwi Sarwani Sri Rejeki, E. Elsa Herdiana Murhandarwati

**Affiliations:** 1 Centre for Tropical Medicine, Faculty of Medicine, Public Health and Nursing, Universitas Gadjah Mada, Yogyakarta, Indonesia; 2 Department of Biostatistics, Epidemiology and Population Health, Faculty of Medicine, Public Health and Nursing, Universitas Gadjah Mada, Yogyakarta, Indonesia; 3 Department of Public Health, Faculty of Medicine, University of Mataram, Mataram, Indonesia; 4 Eijkman–Oxford Clinical Research Unit, Jakarta, Indonesia; 5 Disease Prevention and Control, District Health Office of Kulon Progo, Kulon Progo, Yogyakarta, Indonesia; 6 Department of Public Health, Faculty of Health Sciences, Universitas Jenderal Soedirman, Purwokerto, Central Java Indonesia; 7 Department of Parasitology, Faculty of Medicine, Public Health and Nursing, Universitas Gadjah Mada, Yogyakarta, Indonesia

**Keywords:** Malaria control, Cross-border, Implementation research, Qualitative research, Indonesia

## Abstract

**Background:**

Malaria remains a significant public health issue in Indonesia. Most of the endemic areas are in the eastern parts of Indonesia, but there are a few remaining foci of persistent endemic malaria in Java, particularly in Menoreh Hills, a region bordering three districts of two provinces on this island. Despite a commitment to build a partnership to eliminate cross-border malaria, there is a lack of understanding of how this partnership might be translated into an implementable strategic plan. The study aims to provide evidence of how a participatory approach was used to strengthen the cross-border collaboration and stakeholders’ capacity to develop a joint strategic, operational, and costing plan for cross-border malaria elimination.

**Methods:**

A participatory action research was conducted from January to August 2017, involving participants from the village, district, provincial, and national levels. This study was conducted in seven phases, including document review, focus group discussions (FGDs), planning and costing workshops, and a dissemination meeting. A total of 44 participants from primary health centres (PHC) and 27 representatives of affected villages in three districts, 16 participants from the district and provincial malaria programmes and planning bureaus, and 11 participants from the national level were involved in the processes. Data on priority issues, costing, programme coverage, and administration were collected. Thematic coding and feedback were used for analysis.

**Results:**

Problems identified by stakeholders included low community awareness and participation in malaria prevention, high mobility across three districts, lack of financial and human resources, lack of inter-district coordination, and poor implementation of migration surveillance. Cross-border strategies identified to address malaria were improving cross-border migration surveillance, strengthening the network, governance, and advocacy of malaria control implementation across borders, and developing the malaria information system. A working group composed of the three districts’ representatives authorized to decide on cross-border issues will be created.

**Conclusions:**

The participatory approach was applicable in cross-border malaria planning for within-country settings and useful in enhancing stakeholders’ capacities as implementers. While done in a participatory way, the joint plan crafted was a non-binding agreement; stakeholders should advocate to ensure adequate funds are poured into mobilizing the programme.

## Background

Malaria remains a significant public health challenge in Indonesia. All *Plasmodium* species have been reported in Indonesia, including the new emerging *Plasmodium knowlesi* [1–6]. Nearly half of the 250 million Indonesian population live in malaria-endemic areas and are at risk of malaria infection. It is estimated that in 2017, between 1.2 and 2.0 million people were infected, and around 2700 cases were fatal [7]. Despite the recent success in decreasing the malaria burden from annual parasite incidence of 2.89 per 1000 in 2007 to 0.9 per 1000 in 2017, approximately 60% of total districts and cities have been declared as malaria-free areas [8].

**Fig. 1:**
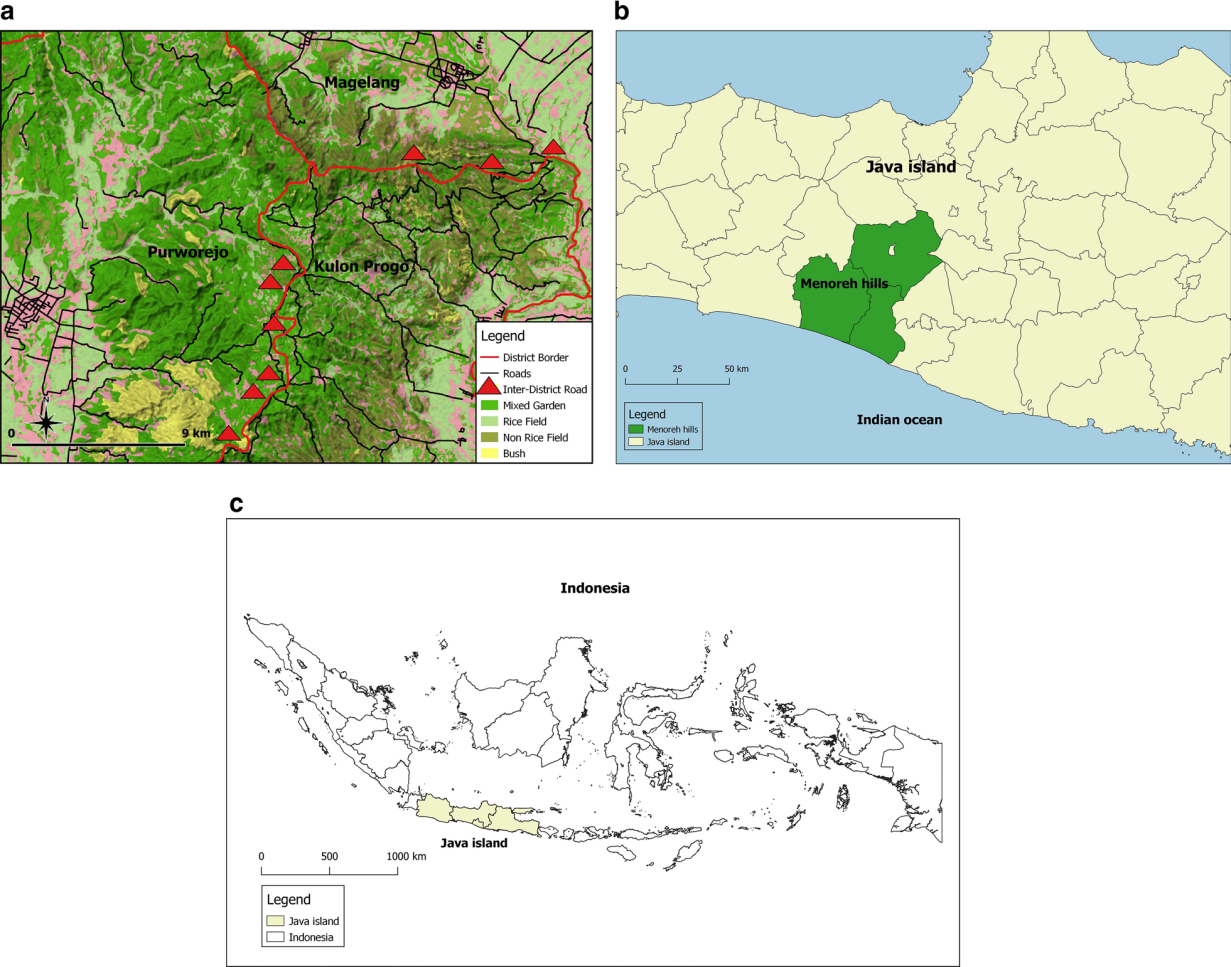
Study site location in the Menoreh Hills Region (Kulon Progo, Purworejo and Magelang District) (a), Central Java, Java Island (b), Indonesia (c)

The malaria-endemic regions in Indonesia are concentrated in eastern Indonesia and some parts of the other large islands. However, in Java Island, which is home to 70% of the population, a few remaining foci of persistent endemic malaria remain. Menoreh Hills, which borders three districts across two provinces, i.e., Kulon Progo District in Yogyakarta Province, Purworejo, and Magelang Districts in Central Java Province, is one of these areas (Fig. 1). The surrounding hills provide an environment favoured by malaria vectors for breeding and resting [9]. Although Magelang has been certified as a malaria-free district, Kulon Progo and Purworejo are still struggling to achieve the status. Menoreh Hills holds one of the highest numbers of malaria cases in Java due to these unique, ongoing situations.

In 2017, 12,512 cases of *Plasmodium falciparum* and *Plasmodium vivax* malaria were recorded from all sub-districts in this region, resulting in an Annual Parasite Incidence (API) 4.0 per 1000 population. Approximately 716,477 people in Purworejo, 1,279,625 in Magelang, and 425.758 in Kulon Progo remain at risk of malaria infection (about 1.6% population of Java Island) [10]. Malaria seroprevalence was reported to be higher in adults, males, and forest workers involved in coconut/palm tapping, fruit farming, logging, and other related jobs [10, 11].

Malaria transmission is facilitated by the high mobility of residents between the affected neighbouring districts (e.g., daily inter-district traditional fruit farming and trading, cultural and religious events that often occur in the night). The inter-district movements are mainly facilitated by multiple two-way main roads (as indicated by red triangles shown in Fig. 1) connecting these three districts. In addition, high-risk groups such as farmers and loggers are very likely to cross the border through a network of small dirt roads crisscrossing villages, fields, and forests when working. Additionally, there are regular migrant workers from malaria-endemic areas outside Java Island who visit their families during the annual religious holiday [12].

Disease control activities to reduce malaria transmission have long been initiated by the local health authorities from all three districts [8, 13]. However, there is a considerable discrepancy in the existing malaria control programme capacity and budget allocation between districts, partly due to the decentralization in the health sector. Therefore, each district also has different strategies in gaining potential funding for malaria control activities.

Following the political decentralization reform in 1999, the health services were decentralized to provincial and district governments. As a result, the Ministry of Health (MoH) decentralized most of the responsibility for planning and service delivery to local governments. However, the relationship between the MoH, the provincial health office (PHO), and the district health office (DHO) is not a hierarchical one. The district government is not a sub-ordinate of the provincial government as most of the power is decentralized to the district government. Additionally, both provincial and district governments operate under the Ministry of Home Affairs [MHA] [14]. As a result, inter-district collaboration systems in malaria to control activities, particularly between provinces, are weak to non-existent. Efforts to address the inter-district migration are scant [13].

Although there has been a commitment from all three districts to build partnerships to eliminate malaria in the region, there is a lack of understanding of how this partnership might be translated into an implementable action plan. There is also confusion about each district authority’s role and responsibilities, not only during routine programme implementation but also during outbreak response [13], especially in Indonesia’s decentralized health system. Therefore, there is a need to strengthen cross-border collaboration in malaria control between neighbouring districts to achieve malaria elimination.

The first step in harmonizing cross-border malaria control in the area would be to create a joint strategic, operational, and costing plan to which all parties ascribe and promise to implement. The World Health Organization ‘Open Skåne 2030’ [15] regional development strategy in Europe has recognized a need for a participatory approach to tackle health inequities and ensure social sustainability, especially in developing countries (Table 1). Although there was ample evidence of participatory action research [PAR] being used in disease control programmes in developing countries in Asia, Africa, and Central America, this approach was used mainly to develop community-based interventions [16–19]. The PAR approach has been demonstrated to effectively create joint strategic plans to eliminate and control malaria in cross-border areas, such as in Greater Mekong Subregion (GMS) [20]. However, literature demonstrating whether this is also applicable in within-country regions that exist as multiple “disconnected” territories because of decentralization policies is limited [21, 22].

**Table 1:** Key messages of *The Open Skåne 2030* Framework ^12^

Key messages
1. Find a common purpose for stakeholders and emphasize the potential of the common good.
2. Focus on the process rather than the product. Create ownership and involvement from all stakeholders.
3. Trust the process. Guide the process by being receptive and owing with rather than controlling it.
4. Emphasize governance processes involving people and power over constructing a formal framework of structures.
5. Create ownership of the process through leadership and ambassadors.
6. Involve and empower other sectors (not only health).
7. Joint mobilization requires leadership characterized by courage and a willingness to take risks.
8. Do background analysis first in order to assess the characteristics of the situation.

This study aims to provide evidence of how a participatory approach can be used to strengthen the cross-border partnership. Particularly, to show how to improve stakeholders’ capacity to develop a joint strategic, operational, and costing plan geared towards eliminating malaria in an area whose boundaries are administratively governed by three districts and two provinces in Indonesia.

## Methods

### Setting

The study was conducted in three districts in the Menoreh Hills Region: Kulon Progo District in Yogyakarta Province, as well as Purworejo and Magelang Districts in Central Java Province, Indonesia, in 2017. Routine DHO surveillance data suggested that 14 sub-districts located in the adjacent areas were affected by malaria. These subdistricts with 1.1 million people were served by 18 PHCs (primary health centres) [23].

As part of the decentralization in the health sector, disease control programmes are managed at the DHO through a network of PHCs and one or more district hospitals depending on the population’s size [24]. Inter-district coordination requires facilitation from PHOs, while inter-province coordination requires facilitation from the MoH. Each level of the local government has its mandates and areas of authority. These arrangements create coordination challenges for joint inter-district collaboration, particularly for bordering districts between two different provinces.

### Design

This study used a mixed-methods study combining secondary data collection and PAR methods involving four sets of participants from different health system levels (village, district, provincial, and national). These participants were key stakeholders who play essential roles in malaria control programmes: the national and subnational malaria control programmes (i.e., communicable disease and malaria control programme managers from MoH, PHO, DHO, and PHC), health service providers (i.e., head of PHCs), other government bodies (i.e., district planning agencies) and representatives of the local communities (i.e., head of malaria-endemic villages, village malaria workers). The government stakeholders were identified from the organogram and discussion within the research team. One co-author was the disease control programme manager at Kulon Progo DHO (TBR), who liaised the research team with the government stakeholders in other districts. Each DHO sent invitation letters to heads of PHCs in endemic subdistricts to participate in the group discussions, who subsequently sent invitation letters to leaders of endemic villages in their catchment areas.

### Data collection and analysis

Data were collected through iterative processes that involved stakeholders from different levels and sectors. During all phases, typed-written notes documented the meetings; group worksheets were collected and summarized into the main worksheet. The list of priority problems identified in Phase 2 were categorized into broadly pre-determined themes, i.e., health services or programmatic, community, and geographical factors using a simple Word processor. The list of strategies and activities identified in Phase 3 was also categorized into broad strategies according to the national malaria control strategies plan using a simple Word processor.

The Softcopies of the voice recordings of the FGDs with stakeholders in Phase 7 were transcribed verbatim using InqScribe Software and then translated to English. AF and TRS independently read the transcripts line by line to identify important themes and quotations. Coding and theme discrepancies were discussed within the group to solve possible disagreements. The findings categorized by themes in each phase were presented during subsequent workshops or consultations for clarification or exploration of additional possible issues. These processes ensured the saturation of the collected data.

Descriptive and summary statistics were done to process the quantitative data for the costing activities in Phase 5.

This study was conducted in seven phases: (1) scientific literature and administrative data review, (2) group discussion using nominal group techniques (NGT) with malaria managers and head of PHCs to identify priority problems in malaria control, (3) joint consultation with DHO staff to prioritize problems and formulate intervention, (4) costing workshop for the strategic and operational plan involving representatives from villages, PHCs and DHO, (5) joint consultation with national and provincial stakeholders about the operational plan including the results of the costing study, (6) finalization of the joint strategic and operational plan with costing study, and (7) dissemination to stakeholders and FGDs to explore the adoption of the strategic plan document. Table 2 summarizes the study phases and the stakeholders involved in each phase.

**Table 2:** Study phases and stakeholders involved in activities

Phase	Participants and organizational affiliations (number of participants)
Phase 1 Literature and secondary data review	Research team
Phase 2 Consultations (agenda: prioritize problems and formulate interventions)	Head of primary health centre (3)
	The staff of the primary health centre (37)
Phase 3 Consultations (agenda: identify priority problems in malaria control)	Head of Planning and Financing Division of Provincial/District Health Office (1)
	Head of Communicable Disease Control Division or Head of Communicable Disease Section Provincial/District Health Office (1)
	Malaria programme manager from each district (3)
Phase 4 Consultations (agenda: costing workshop with community representatives)	The staff of the primary health centre (44)
	Village representatives (27)
Phase 5 Consultations (agenda: consultation with national and provincial stakeholders)	National level: Ministry of Health (Malaria sub directorate and vector-borne disease directorate) (6), Expert Committee of Malaria (3), Centre for Environmental Health and Engineering (1), and Centre for Vector Research (1)
	Provincial-level: PHO (2), Provincial Planning Bureau (2), District Revenue Bureau (2), local government representatives (2)
	District level: DHO (4), District Planning Bureau (2), local government representatives (2)
Phase 6 Strategic plan document writing	Research team members
Phase 7 Dissemination of the strategic plan	All stakeholders who were participated in the development of the strategic plan (Phase 2–5) were invited to attend the dissemination meeting.

Before primary data collection, two key malaria control stakeholders from each health office (three DHOs and two PHOs) were recruited as facilitators. They were intensively trained to systematically facilitate each step of the joint strategic and operational planning and costing for malaria elimination. During the consultations (Phase 2–5), these trained facilitators performed group discussions and brainstorming sessions with guidance from study investigators.

In Phase 2, six group discussions to explore priority problems in three districts were conducted with 40 participants from PHCs with high malaria endemicity. Group discussions were conducted separately with head and malaria programmers of PHCs. The NGT was used to elicit priority issues in malaria control. NGT is an effective method to ensure relatively equal participation, is a time-saving technique, and can produce many ideas while avoiding unnecessary conflict. The NGT has four steps in which (1) everyone in the group generates and writes down their ideas; (2) group members take turns to record each idea on a board concisely; (3) each recorded idea is discussed to obtain clarification; then, (4) each individual votes privately on the priority of the ideas. A group decision is made based on the ratings. Notes were taken during the discussions [25]. Audio-recordings were taken. However, as the recordings were not clear, only notes and worksheets were analysed.

In Phase 3, a two-day consultation meeting was conducted involving malaria programmers and communicable disease control managers from the three districts and two provinces. A total of 13 people participated in the meeting. During the workshop, the study investigators presented the priority problems identified during the group discussions in Phase 2 and its strategies. Participants discussed and formulated the following components of the action plan: priority problems at district and cross-border level, vision, mission and objectives, targets and indicators, strategies and activities, a monitoring and evaluation framework, and governance.

Costing exercises were conducted in Phase 4, involving 44 malaria staff from PHCs and 27 village representatives. Pre-structured worksheets were used to guide the planning and budgeting exercise. The worksheets consisted of categories of technical strategies taken from the national malaria strategic plan. They also consisted of budget categories that were used in disease control programme planning. In addition, administrative data (from present and previous years), price quotations from suppliers, guidelines, and policy documents were also collected to approximate the monetary value of the joint plan’s identified needs for collaborative malaria control activities. The village representatives were consulted about exploring the possibility of organizing and funding community-based activities through the existing "dana desa". Dana Desa is a government funding scheme that was disbursed to the village level for various community-based development projects.

In phase 5, consultation meetings with the district, provincial, and national stakeholders were organized. The meeting was attended by district and provincial malaria managers, district and provincial planning bureaus, MoH representatives, malaria experts, and other relevant stakeholders (Table 2). They were consulted to harmonize the proposed technical strategies according to the national malaria strategic plan. Implementation strategies were also discussed.

All the findings from Phase 2–5 were incorporated into the strategic and operational plan documents during Phase 6. Data collected from scientific literature in cross-border malaria abroad were used as complements to guide the initial draft of the strategic, operational, and costing plan. The study investigators developed the planning document through an iterative consultation process with the district and provincial health offices’ facilitators. Feedback from the DHOs and PHOs were incorporated to revise further and refine the document. All DHOs and PHOs approved the final document.

Phase 7, after all districts and provinces agreed with the documented strategic and operational plan, a dissemination meeting was conducted. All relevant stakeholders who were involved in the development process were invited. The meeting was also used to obtain an endorsement from the national malaria control programme. At the end of the dissemination meeting, we organized FGDs with the meeting participants to explore the acceptance and the best way to adopt the strategic and operational plan into district and provincial policies.

## Results

### Problem priorities and strategies

Priority problems identified by participants during FGDs in Phase 1 included (1) health service aspects: lack of financial and human resources, lack of inter-district coordination between PHCs and DHOs, insufficient endorsement of migration surveillance policy, and limited role of local government; (2) community aspects: low community awareness and participation in malaria prevention, and high mobility of residents between districts, and (3) geographical challenges: rugged terrains and too many breeding sites along most of rivers or streams in the area. Table 3 summarizes the priority problems identified in Phase 2 and confirmed in Phase 3.

**Table 3:** Problems Identified during Phase 2

Problem	Magelang	Purworejo	Kulonprogo
*System*			
Funding (amount and budgeting mechanism)	✓	✓	–
Intersectoral coordination	✓	✓	✓
Current regulation at village and sub-district level	✓	✓	✓
Community malaria workers (inadequate numbers or skills)	✓	✓	✓ (inadequate in skills)
Health care workers (inadequate in numbers or skills)	✓	✓	–
Lack of stakeholders’ commitment (leadership)	✓ (District)	✓ (District, Sub-district, Village)	✓ (Village)
Lack of infrastructure and facilities (Labs, consumable, RDT, Bednets)	✓	✓	–
Confusion about case definition among health care workers (import vs. indigenous), particularly in cross-border villages	✓	–	✓
*Community*			
KAP is low: Perceived fear from malaria Treatment adherence Community participation in migration surveillance is poor	✓	✓	✓
High cross-border mobility	✓	–	✓
Imported cases from outside Java Island	✓	✓	✓
*Environmental*			
Difficult geographical accessibility	✓	✓	✓
Breeding place along many rivers	✓	✓	✓

Additionally, funding mechanisms and cycles were considered a potential problem for implementing the proposed strategic plan. Phase 2 to Phase 6 were conducted between April and September 2017. However, the funding planning cycle for 2018 had already been closed before the strategic plan was finished.

“Although we really would like to execute this action plan as soon as possible to achieve elimination in 2021, our funding cycle for 2018 has been closed, so we have to propose these activities in the 2019 district budget.“

(District manager).

During Phase 3, three strategies were identified as the joint operational plan pillars: (1) strengthening early detection and rapid response systems, (2) addressing malaria focus through local mass approach and vector control, and (3) ensuring availability and access of diagnosis and management for malaria cases.

Both participants from Phase 2 and 3 also identified the following strategies to address cross-border malaria: (1) intensifying population migration surveillance, (2) strengthening networking, governance, and advocacy of the implementation of malaria control across borders with relevant stakeholders, including cross-sectoral, and (3) the development of a malaria information system in Menoreh Hills area. Furthermore, malaria managers from three districts agreed to create a working group composed of representatives from the three districts authorized to address cross-border issues related to malaria. They also identified four major funding allocations required: indoor residual spraying, distribution of long-lasting insecticide-treated nets, diagnosis and treatment, and surveillance and reporting.

The proposed intervention strategies, activities, and the corresponding costings were presented and consulted during the national meeting in Phase 5. The national meeting’s objective was to harmonize and refine the proposed strategies with the national malaria strategic plan, taking into account national malaria stakeholders’ inputs. Inputs gathered during the discussion include revision of district activities based on effectiveness and relevance, revision of costing based on the number of foci, and further clarifying roles of the district, province, and national stakeholders.

### Strategic plan

The 5-year Malaria Elimination Action Plan in Menoreh Hills was developed. Inputs and feedback from various stakeholders covering all health system levels were used to shape the document. The action plan was approved by stakeholders from the three districts and the MoH Indonesia. This joint action plan covers detailed information on the settings’ baseline profiles, strategic issues, vision, mission, and goals for malaria elimination in the region, malaria elimination strategies, costing and budgeting, implementation and governance, and monitoring and evaluating the 5-year Malaria Elimination Action Plan. The joint action plan document will then be implemented as local-specific guidance for malaria elimination activities in the region.

As part of the Malaria Elimination Action Plan, a joint task force to implement and evaluate the joint operational plan was proposed. The national malaria control programme supports this proposal via the MoH Indonesia Decree (KepMenKes RI No HK.01.07/Menkes/498/2017). The joint task force is responsible for: (1) decision making and inter-district coordination, (2) programme planning, monitoring, and evaluation, (3) establishing information systems in the region, (4) technical activities planning and implementation, and (5) data validation and synchronization. Furthermore, a technical officer will assist with three main areas: epidemiology, entomology, and community engagement. The technical officer is responsible for: (1) assisting the execution of decisions related to programme implementation made by the joint task force, (2) acting as an information clearinghouse to accommodate and ensure information is regularly shared between the three districts, (3) validating and synchronizing data for monitoring and evaluation purposes, (4) ensuring programme planning, monitoring and evaluation are periodically performed, and (5) providing technical support for the malaria elimination programme in the three districts. The technical officer is hired by and reports to the national malaria programme for the period of implementing the strategic plan.

### Acceptance and adoption

Participants mentioned that the action plan is urgently needed to achieve malaria elimination in the area by 2021. Malaria elimination has been included as a district’s development indicator and provincial mid-long term development plan. To be operationalized, the action plan needs to be anchored with the district’s and province’s mid-long term development plan to allow funding allocation. It is also important to synchronize the action plan with the district’s funding cycle, as previously mentioned by participants when discussing problems for implementing the strategic plan.

In terms of costing and budget planning for the action plan, it was agreed that all levels should be involved to ensure adherence to the funding guideline.

“The national budget already has their menu items for activities, but some of the activities in this action plan are not listed. So, the MoH needs to join the budget discussion because they will know what is needed. For the local budget, it is more flexible.“

(provincial manager).

Although most participants recognized the importance of the research process in informing the development of the action plan, there was a difference in the timeline between the research process and development milestones.

“Academics have to undergo a long process – these might indeed be the necessary steps, but as practitioners, we wanted a quick result.“

(provincial manager).

“While developing the mid-long term development plan, we had to develop a strategic plan within one month, so this action plan will not be able to catch up.“

(provincial manager).

Participants also agreed that involving stakeholders from other sectors is essential, and for this, establishing a legal basis for the action plan execution is very important.

“There should be a regulation from the District Head or the Governor to support the action plan, and in there, the roles of each government unit of other sectors will be specified. Each should determine their target. Since this involves more than one province, there should be a joint decree from both Governors for the districts.“

(Provincial manager).

Participants also agreed that tackling cross-border malaria requires joint commitment and collaboration.

## Discussion

This study showed that the participatory approach’s applicability to tackle cross-border malaria in an in-country area whose jurisdiction is shared by three districts and two provinces within a decentralized health system. As was done by the World Health Organization in the GMS [20], a bottom-up approach was used to involve key stakeholders, including those in the health systems’ lower levels. This approach has enabled identifying problem priorities and potential strategies in accelerating elimination that is unique to the region. It has been reported that locally tailored control strategies are essential for malaria elimination in Indonesia due to the high levels of decentralized authority [13]. Most importantly, this study created a joint strategic, operational, and costing plan to address cross-border malaria in the Menoreh region.

The issue of cross-border malaria is a critical issue that hampers the elimination of malaria globally. It has been made more difficult because of logistical and political technicalities in areas that are considered shared territories. The findings of this study suggest that difficulties of cross-border malaria control in within-country settings are similar to challenges encountered in inter-country settings. Different programme capacities, people mobility across borders, lack of joint planning and coordination, and inadequate cross-border surveillance and response were among the main themes identified [26–29]. Likewise, solutions enumerated by stakeholders were also identical to those suggested for international cross-border settings [29].

The study adds to the body of evidence as to how participatory action research methods can be used for health planning in vector-borne diseases. While the participatory approach has been previously used in inter-country cross-border malaria, this study demonstrated that this can also be useful in within-country settings that are seemingly disjunct because of decentralization policies. Indeed, programme managers and stakeholders’ participation have been found to stimulate local capacities and potentially spur sustainable interventions [16]. The next steps would be to roll-out participatory or community-based interventions as done elsewhere [17, 18].

## Study limitations

Although we used a participatory approach where everyone should have the same opportunity to contribute their views, participants in the FGDs and workshops had hierarchical relationships at work. This relationship could create reluctance among some participants to express their views. However, to compensate for this limitation, conversation with different participants between group discussion sessions was used to explore different opinions. There were many malaria control ideas proposed by participants from the PHCs and DHOs/PHOs staff, for example involving local artist and traditional art to mobilize the community. However, they were restricted by the strict reporting guidelines, especially on financing.

Mismatch in the timeline between programme and research was one of the barriers encountered during the process. While the action plan must be produced within a short timescale, the researchers had to undergo several steps to deliver the action plan. This is a common problem in the effort of linking research and policy [16].

## Conclusions

This study found that the participatory approach involving key stakeholders is applicable in coordinated inter-district malaria planning. The participatory approach facilitated joint priority settings and joint programme planning, including sharing resources and budgeting, which was not feasible in the routine health system. Stakeholders found this approach to be useful, especially in enhancing their capacities as implementers. While done in a participatory way, the joint action plan crafted is a non-binding agreement; stakeholders should advocate to ensure that adequate funds are allocated so that plans can be mobilized. Studies to identify bottlenecks in implementation fidelity are suggested. Future studies should also consider the quantification of within-country, cross-border movement as additional evidence to further guide elimination strategies.

## Data Availability

The datasets used and analyzed during this study are not publicly available due to the inclusion of identifying information on individuals but are available from the corresponding author on reasonable request.

## Abbreviations

DHO: District health office
FGDs: Focus group discussions
GMS: Greater Mekong Subregion
MoH: Ministry of Health
PAR: Participatory action research
PHC: Primary health centre
PHO: Provincial health office
